# The effect of personalized forest therapy programs on stress and obsessive-compulsive symptoms in patients with depression

**DOI:** 10.3389/fpubh.2025.1735731

**Published:** 2026-01-12

**Authors:** Gayeon Kim, Sinae Kang, Kyungsook Paek, Youngeun Seo, Seyeon Park, Hyoju Choi, Gyeongmin Min, Neeeun Lee, Sooil Park, Saeyeon Choi, Pyeongsik Yeon

**Affiliations:** 1 Graduated Department of Forest Therapy, Graduate School of Chungbuk National University, Cheongju, Republic of Korea; 2 Department of Forest Sciences, Graduate School of Chungbuk National University, Cheongju, Republic of Korea; 3 Department of Forest Sciences, Chungbuk National University, Cheongju, Republic of Korea

**Keywords:** diagnostic evaluation, healthcare, mental health, non-pharmacological therapy, prescription, urban forests

## Abstract

**Introduction:**

Depression is one of the most serious mental health disorders worldwide. Forest therapy has been shown to be an effective non-pharmacological intervention. This study examined the development and implementation of personalized forest therapy programs for patients with depression, particularly their effects on stress levels and obsessive-compulsive symptoms.

**Methods:**

Participants included 29 adults aged 20 to 50 years who had been diagnosed with mild depressive disorder. Using a randomized controlled trial design, the participants were divided into two groups: an experimental group (*n* = 17) and a control group (*n* = 12). The experimental group was provided with a personalized forest therapy program developed based on individual characteristics, while the control group continued with their usual medication and counseling treatment. The program was conducted once a week for 120 min over 4 weeks at Seoul Forest. Stress and obsessive-compulsive symptoms were measured using the Perceived Stress Scale (PSS) and the Maudsley Obsessive-Compulsive Inventory (MOCI), respectively.

**Results and Discussion:**

The results showed that personalized forest therapy programs reduced both stress levels and obsessive-compulsive symptoms in the patients with depression. These findings suggest that personalized forest therapy programs could have a positive impact on the mental health of patients with depression and provide scientific evidence to support their integration into the healthcare sector.

## 1 Introduction

Depression is recognized as a major global mental health concern. According to the World Health Organization (1), approximately 280 million individuals worldwide are affected by depression, representing approximately 3.8% of the global population. As with other mental health disorders, depression has been linked to an increased risk of reduced motivation, substance dependence (e.g., alcohol), and, in extreme cases, suicide (2). Among individuals with suicidal ideation, the prevalence of depressive disorder is 60.1%, followed by anxiety disorder (33.4%) and alcohol use disorder (32.4%). Therefore, depressive disorder is the most prevalent condition linked to suicide, followed by anxiety disorder (3), contributing to adverse outcomes and substantial impairment in quality of life.

Individuals with depressive disorders experience not only core depressive symptoms but also an extensive array of additional manifestations, including sleep disturbances, anxiety, decreased concentration, fatigue, and anhedonia (4). Depression is also associated with psychosocial factors, as individuals with elevated levels of stress or a low degree of resilience are more prone to developing depressive disorders (4).

The relationship between stress and depression has been a subject of discussion for some time. A clear directional association has not been established, as depression can heighten stress and stress can exacerbate depression. However, research has demonstrated that a relationship between them exists (5). Stress originates from various factors, including professional obligations, academic pursuits, and interpersonal dynamics. Severe or chronic stress has been shown to deplete mental and physical resources, leading to a state of exhaustion (6). It can also act as a catalyst for behaviors and emotions associated with depressive and anxiety disorders. Prolonged excessive cortisol secretion, a consequence of stress, may further induce neurological dysfunction (7).

Depression is often comorbid with obsessive-compulsive disorder (OCD). According to extant research, 13–75% of patients with OCD also have depressive disorders, making depression the most common comorbidity (8). The American Psychiatric Association (9) defines OCD as a disorder in which intrusive thoughts or impulses repeatedly enter a person’s consciousness against their will, leading to obsessive fixation and the repetition of related behaviors. Individuals with OCD often experience adverse emotions, including anger, anxiety, and depression, attributable to their perceived inability to meet external expectations and standards while recognizing that they cannot control these standards (10). Accordingly, the management of obsessive symptoms and stress associated with depressive disorders must be integrated into depression treatment strategies.

The treatment of depressive disorders is influenced by several key factors, including suicide risk, psychosocial functioning, psychosocial stress, and the presence or severity of functional impairment (11). Available treatment options range from pharmacological therapy and psychotherapy to electroconvulsive therapy, with growing recognition of the need to integrate pharmacological and non-pharmacological approaches (12). Among non-pharmacological methods, forest therapy has garnered increased interest, accompanied by a rise in related research endeavors. Forest therapy is a practice that promotes physical and psychological well-being by engaging the five senses with natural stimuli, including scenery, sounds, and scents, in forest settings. A meta-analysis by Rosa et al. (13) found that forest therapy is more effective in alleviating depressive symptoms than treatment conducted in hospitals or urban areas. Townsend (14) further suggested that participants in forest-based interventions experienced reduced fear of natural environments and enhanced appreciation of nature, as well as increased relaxation, self-confidence, and empathy through shared experiences with others.

Evidence has consistently demonstrated the efficacy of forest therapy in alleviating symptoms of depression, thereby underscoring the necessity for collaborative efforts with the healthcare sector. For instance, Zhang et al. (15) observed that forest therapy can offer effective solutions to public health issues by delivering multiple health benefits, stressing the importance of strengthening collaboration with the healthcare sector. This approach requires both objective evidence and a systematic framework for implementing forest therapy. Yeon et al. (16) suggested that research is needed to identify optimal forest characteristics and structured activities for reducing depressive symptoms. In response to this need, Kim et al. (17) analyzed programs across various fields aimed at alleviating depression, anxiety, and stress and classified 59 therapeutic activities into two categories—purposeful activities and practical (utility) activities. They emphasized that these activities should be delivered according to the characteristics of each participant. The prevailing discourse is that, rather than implementing comprehensive forest therapy programs for general audiences, scholars advocate tailoring activities to specific participant groups (18).

The importance of delivering interventions that reflect individuals’ characteristics and contextual circumstances has been emphasized across multiple disciplines, including medicine, psychology, and education. In medicine, the concept of precision medicine highlights the need to tailor interventions based on personal attributes and environmental factors. Similarly, personalized treatment in psychology and personalized learning in education underscore the value of individualized approaches that incorporate a participant’s unique profile.

In the field of forest therapy, personalized approaches have also gained traction through the introduction of nature prescription systems. In the United States, physicians, health coaches, and nurse practitioners prescribe time in natural environments or nature-based physical activities to their patients (19). In the United Kingdom, a national, healthcare-linked system collaborates with community organizations to provide various nature-based activities, such as physical exercise and horticultural activities (20). These nature prescriptions have been shown to exert beneficial effects on mental health—including depression, anxiety, and stress (21)—as well as on physiological outcomes such as blood pressure and physical activity levels (22).

Although several countries, including the United States, the United Kingdom, Germany, and Canada, have begun implementing nature prescription models that integrate healthcare systems and provide individualized nature-based interventions, most prescriptions remain general rather than fully tailored. Therefore, the current study emphasizes the need for a personalized approach in forest therapy, in which healthcare providers determine program components that align with each patient’s individual characteristics and clinical condition. Based on these tailored recommendations, forest therapy practitioners can then develop and deliver personalized intervention programs.

Based on this rationale, this study aimed to develop and implement personalized forest therapy programs for patients with depression and to examine changes in their levels of stress and obsessive-compulsive symptoms. Forest therapy activities were designed in consultation with patients’ attending specialists to ensure alignment with individual needs. Programs were then developed based on these considerations, and their effectiveness and feasibility were evaluated through empirical implementation.

## 2 Materials and methods

### 2.1 Study design

This study employed a randomized controlled trial design comparing an experimental group (personalized forest therapy program) with a control group (usual care). After randomly assigning participants to either group, the experimental group was further divided into two subgroups based on the diagnosis of the attending specialists. Following the group assignment, a pre-test was conducted from 21 May to 24 May 2025. Subsequently, the experimental group was divided into two teams, and a forest therapy program was implemented for each team. A post-test was conducted from 15 June to 21 June 2025, coinciding with the end of the program. The control group continued their usual medication and counseling regimens. Upon completion of all programs for the experimental group and the administration of post-tests for both groups, the control group also received one session of forest therapy.

### 2.2 Participants

Participants were recruited from three psychiatric departments in Seoul, South Korea. Psychiatrists at each institution identified patients who were deemed suitable for participation in forest therapy and directly assessed their willingness to participate. The eligibility criteria were as follows: (a) adults aged 20–50 years diagnosed with mild depressive disorder by a psychiatrist according to the DSM-5 criteria, (b) the ability to engage in outdoor activities for 2 h or more, and (c) absence of cognitive impairments that might interfere with participation in surveys or forest activities. This study targeted individuals with a diagnosis of mild depressive disorder; however, it did not assess whether participants had been formally diagnosed with stress-related or obsessive-compulsive disorders. Although 36 individuals initially expressed interest, seven dropped out during the experiment due to reasons such as scheduling conflicts, survey non-compliance, treatment discontinuation, and others, resulting in a final experimental group of 17 participants (five men, 12 women; mean age 37.65 ± 9.79) and a control group of 12 participants (four men, eight women; mean age 34.17 ± 6.44). The final sample consisted of 29 participants (mean age 36.21 ± 8.61). Prior to study initiation, its objectives and methodologies were explained to the participants, and written informed consent was obtained. The participants were blinded to their group allocation, and the control group received a single session of the same program after the experimental group had completed the intervention. Ethical approval was granted by the Institutional Review Board of Chungbuk National University (IRB no. CBNU-2025-A-0034).

### 2.3 Study site

**Figure 1:**
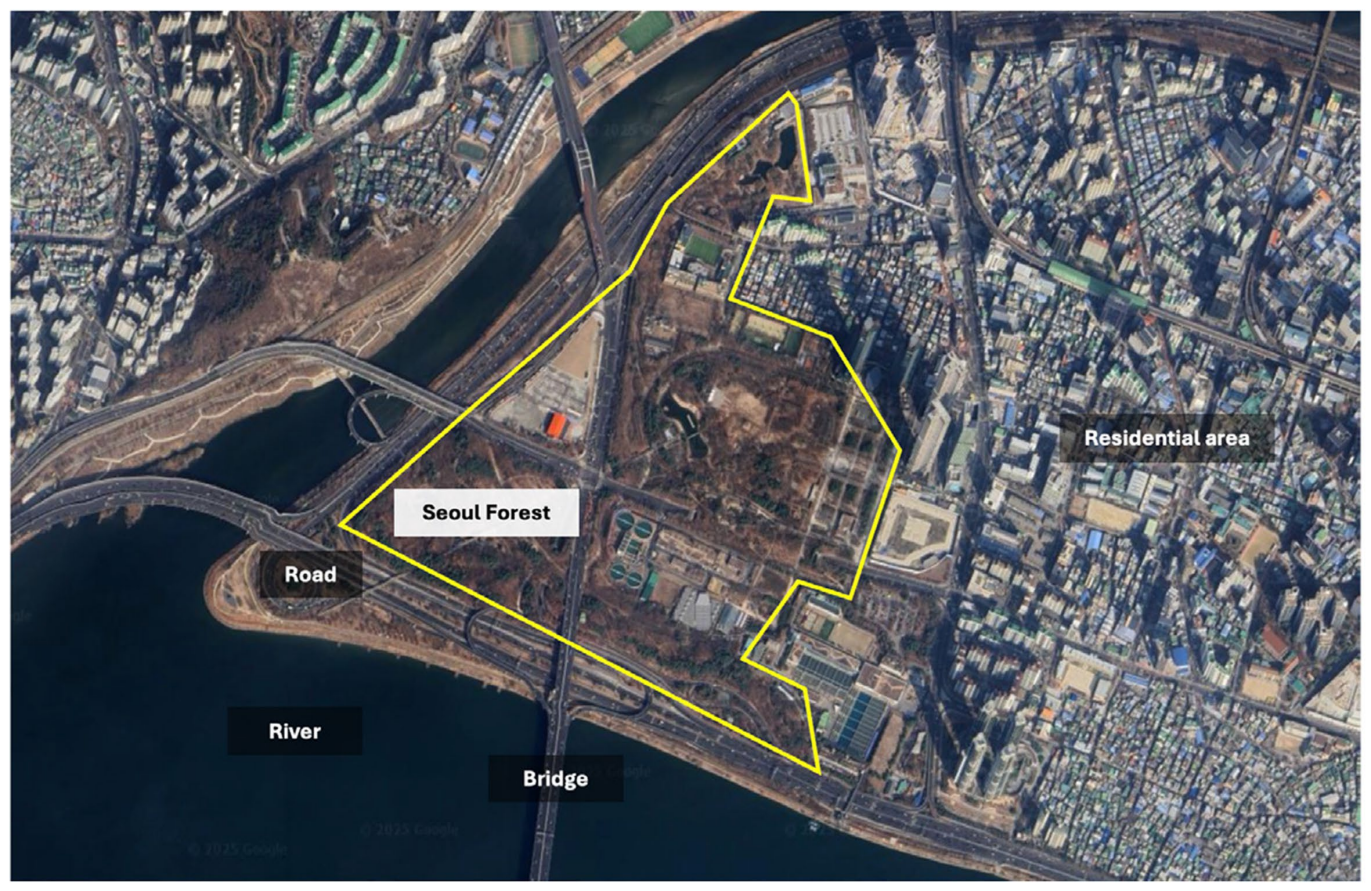
Study site: Seoul Forest (https://earth.google.com/web/@37.54600762,127.03744772,11.61096015a,3936.72192641d,35y,-0h,0t,0r) (Accessed August 4, 2025).

This intervention was conducted at Seoul Forest in Seongdong-gu, Seoul, South Korea (Figure 1). This location was selected for its accessibility and facilities, including proximity to public transportation options (e.g., subway and bus stops) and parking. The forest covers 50 hectares and features diverse amenities such as an outdoor stage, butterfly greenhouse, nature learning center, fragrance garden, Ginkgo forest, walking trails, and restrooms. This urban natural space offers a variety of ecological programs for children and youth, as well as therapy programs, including yoga and meditation, conducted in outdoor settings. The forest includes conifer species (e.g., *Pinus strobus, Pinus densiflora, Ginkgo biloba,* and *Metasequoia glyptostroboides*) and broadleaf trees (e.g., *Prunus serrulate, Cercidiphyllum japonicum*, and *Zelkova serrata*). No precipitation was recorded during the study period, and the mean temperature in Seoul was 20.9 °C.

### 2.4 Personalized forest therapy program

Personalized forest therapy programs were co-developed by each participant’s attending specialist, a certified forest therapy instructor, and the research team. Physicians conducted the primary assessment of the participants, meeting them in person whenever possible; if an in-person consultation was not feasible, information was obtained through a structured telephone interview. During this assessment, physicians selected one of four activity types—“self-understanding and self-management activities,” “sensory stimulation and mind–body relaxation activities,” “physical activities,” and “nature-based and art-mediated activities”—based on their clinical judgment regarding each participant’s needs. In addition, specialists identified relevant participant considerations across nine categories: “allergic reactions,” “uncomfortable body parts,” “sensory sensitivity,” “physical contact sensitivity,” “specific objects,” “specific events and memories,” “self-disclosure,” “open spaces,” and “specific locations.”

Following data collection, physicians documented their recommendations and delivered them to the research team, who then collaborated with the forest therapy instructor to design detailed program content reflecting these individualized considerations. Across the participants, the most frequently selected activity types were “self-understanding and self-management activities” and “sensory stimulation and mind–body relaxation activities,” while two participants required specific modifications related to “physical contact sensitivity.”

Based on the specialists’ recommendations, two personalized forest therapy programs were developed: Program A (implemented for nine participants) and Program B (implemented for eight participants). Program A included “self-understanding and self-management activities,” “sensory stimulation and mind–body relaxation activities,” and “nature-based and art-mediated activities,” whereas Program B included “self-understanding and self-management activities,” “sensory stimulation and mind–body relaxation activities,” and “physical activities.” Although the programs differed in activity structure, they shared the same therapeutic objectives and followed common therapeutic stages: exploration → recognition → change → integration.

**Figure 2:**
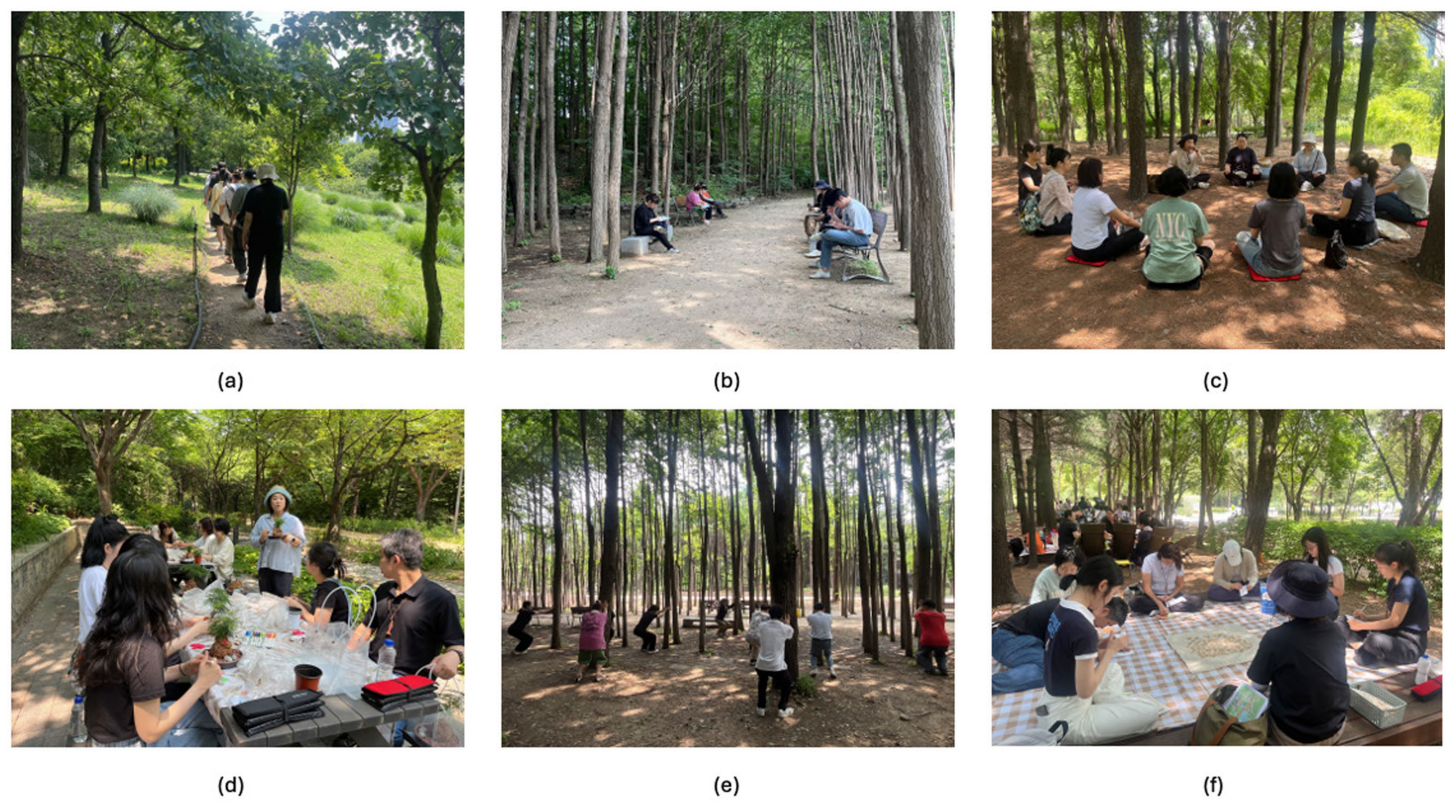
Forest therapy program activities: **(a)** Forest walking; **(b)** self-exploration; **(c)** meditation; **(d)** art; **(e)** physical exercise; **(f)** emotional exploration.

Each participant’s attending specialist reviewed the assigned program activities to ensure that the content was clinically appropriate and that no safety or suitability concerns were present. The final program design considered therapeutic goals, selected activity types, participant-specific precautions, and the environmental and facility conditions of the program site. A certified forest therapy instructor delivered all activities and thoroughly reviewed the program content and individual considerations before implementation. Figure 2 presents the overall structure of the therapy programs, and Table 1 provides a detailed description of the activities.

**Table 1:** Detailed description of the forest therapy programs.

Therapy stage (Session)		Exploration (Session 1)	Recognition (Session 2)	Change (Session 3)	Integration (Session 4)
Activities by program					
Common activities	Icebreaker	Hand pressure point massage	Clapping	Group rope untying	Following guidance with eyes closed
	Activities to awaken the five senses	Engaging all five senses while walking through the forest	Engaging all five senses while walking through the forest	Engaging all five senses while walking through the forest	Engaging all five senses while walking through the forest
	Self-exploration activities	Photographing natural objects that represent me	Finding the “me others see” and the “me others do not see”	Decorating my self-tree (writing what I want to change)	Drawing life’s mountain ranges
	Meditation	Breath meditation + walking meditation			
	Emotion exploration activities	Finding emotions to keep and to let go of	Finding emotions to keep and to let go of	Finding emotions to keep, to let go, and to pursue	Reflecting on emotions so far
Program A additional activities	Art activities	Pressed flower bookmarks and handkerchiefs	Topiaries	Worry dolls	Mobiles
Program B additional activities	Forest walk	Walking correctly	Walking by following the person in front	Walking barefoot	Walking together (using a rope)
	Physical function enhancement exercises	Strength training			

### 2.5 Experimental procedure

Both forest programs were delivered once a week for 120 min over a four-week period. Program A was conducted with nine participants every Sunday (May 25 to June 15, 2025), and Program B was conducted with eight participants every Tuesday (May 27 to June 17, 2025). Both programs were led by the same forest therapy instructor, with two assistant instructors participating in each session. The control group did not participate in forest activities during this period; instead, they continued with standard pharmacological therapy and counseling. The control group received the forest program only after the experimental group had completed the experimental sessions to prevent the participants from knowing whether they were in the experimental or control group.

### 2.6 Measurement scales

This study utilized the Perceived Stress Scale (PSS) and the Maudsley Obsessive-Compulsive Inventory (MOCI) to measure the levels of stress and OCD symptoms in the patients with depression.

Developed by Cohen et al. (23) and validated in Korean by Park and Seo (24), the PSS is a widely used measure of psychological stress. This scale consists of 10 items, each rated on a 5-point Likert scale from 0 (not at all) to 4 (extremely). It is divided into two subdomains: negative perception and positive perception (24). The negative perception subdomain reflects stress arising from negative cognitive appraisal, characterized by perceived lack of control, unpredictability, and feelings of being overwhelmed during stressful situations. In contrast, the *positive perception* subdomain reflects stress resulting from the absence of positive appraisal, such as diminished perceptions of controllability, predictability, and emotional stability, even in stressful contexts. This subdomain is calculated using reverse-scored items, and higher scores indicate a higher level of perceived stress. Scores of 0–13 are considered normal, 14–16 indicate mild stress, 17–18 indicate moderate stress, and 19 or above indicate severe stress.

The MOCI was developed by Hodgson and Rachman (25) and validated in Korean by Min and Won (26) to assess the types and severity of observable obsessive-compulsive behaviors. The MOCI comprises 30 items answered with either “yes” (2 points) or “no” (1 point). A higher score indicates a higher level of obsessive-compulsive behavior. Scores of 30–43 are considered normal, 44–48 indicate a tendency toward obsessive-compulsive behavior, and 49 or higher reflect severe obsessive-compulsive behavior.

### 2.7 Data analysis

Data were analyzed using IBM SPSS Statistics version 29 (Mac). The mean and standard deviation of the data were calculated, and an independent samples *t*-test was conducted on the pre-test scores to verify homogeneity across the groups. Subsequently, a paired sample *t*-test was conducted to compare the pre- and post-test results for each group. The statistical significance level was set at a *p*-value of <0.05.

## 3 Results

### 3.1 Homogeneity test

Prior to comparing pre- and post-test scores, a homogeneity test was conducted between the two groups using an independent samples *t-*test on baseline stress and obsessive-compulsive symptom scores. The results showed no statistically significant differences for either stress (*t* = 0.669, *p* = 0.509) or obsessive-compulsive symptoms (*t* = 0.957, *p* = 0.347), indicating that the two groups were homogeneous at baseline (Table 2).

**Table 2:** Results of the homogeneity test for pre-test scores (independent samples *t*-test).

Variable	Group	*n*	*M*	SD	*t*	*p*
Stress	Experimental group	17	22.88	6.25	0.669	0.509
	Control group	12	21.33	5.97		
Obsessive-compulsive symptoms	Experimental group	17	43.82	4.50	0.957	0.347
	Control group	12	41.92	6.24		

### 3.2 Stress

Paired sample *t*-tests were conducted to assess changes in pre- and post-test stress scores for each group (Table 3). The experimental group, which received personalized forest therapy programs, demonstrated a statistically significant reduction in stress scores (*t* = 2.540, *p* = 0.022, *g* = 0.587). In contrast, the control group showed no statistically significant difference (*t* = 0.809, *p* = 0.436, *g* = 0.217).

**Table 3:** Results of the comparison of total stress scores before and after the forest therapy program (paired sample *t*-test).

Group	Timepoint	*n*	*M*	SD	*t*	*p*	Effect size (*Hedges’ g*)
Experimental group	Pre-test	17	22.88	6.25	2.540	0.022	0.587
	Post-test		18.94	5.88			
Control group	Pre-test	12	21.33	5.97	0.809	0.436	0.217
	Post-test		20.25	7.43			

The results of the paired sample *t*-test, conducted to assess changes in stress scores across the subdomains for each group, are presented in Table 4. In the negative perception subdomain, no statistically significant differences were observed for either the experimental group (*t* = 1.665, *p* = 0.115, *g* = 0.384) or the control group (*t* = 0.774, *p* = 0.455, *g* = 0.208). However, in the positive perception subdomain, the experimental group demonstrated a statistically significant decrease (*t* = 2.395, *p* = 0.029, *g* = 0.553), while the control group showed no significant difference (*t* = 0.545, *p* = 0.596, *g* = 0.146).

**Table 4:** Results of the comparison of subdomain stress scores before and after the forest therapy program (paired sample *t*-test).

Subdomain	Group	Timepoint	*n*	*M*	SD	*t*	*p*	Effect size (*Hedges’ g*)
Negative perception	Experimental group	Pre-test	17	9.71	4.27	1.665	0.115	0.384
		Post-test		7.82	4.02			
	Control group	Pre-test	12	10.83	4.91	0.774	0.455	0.208
		Post-test		10.25	5.55			
Positive perception	Experimental group	Pre-test	17	13.18	3.03	2.395	0.029	0.553
		Post-test		11.12	3.10			
	Control group	Pre-test	12	10.50	2.28	0.545	0.596	0.146
		Post-test		10.00	3.22			

### 3.3 Obsessive-compulsive symptoms

Paired sample *t*-tests were also conducted to examine changes in pre- and post-test obsessive-compulsive symptom scores across both groups (Table 5). The analysis indicated that, while the experimental group demonstrated a decrease in obsessive-compulsive symptom scores, this reduction was not statistically significant (*t* = 1.490, *p* = 0.156, *g* = 0.384). In the control group, obsessive-compulsive symptom scores increased, but the change was not statistically significant (*t* = −1.168, *p* = 0.267, *g* = 0.208).

**Table 5:** Results of the paired sample *t*-test comparing obsessive-compulsive symptom scores before and after the forest therapy program.

Group	Timepoint	*n*	*M*	SD	*t*	*p*	Effect size (*Hedges’ g*)
Experimental group	Pre-test	17	43.82	4.50	1.490	0.156	0.384
	Post-test		42.59	3.55			
Control group	Pre-test	12	41.92	6.24	−1.168	0.267	0.208
	Post-test		42.50	7.45			

## 4 Discussion

This study examined the effects of personalized forest therapy programs on stress and obsessive-compulsive symptoms in patients with depression. The findings suggest that such programs exert positive effects on both outcomes. The main points of discussion are as follows.

First, stress levels in the experimental group decreased significantly following participation in personalized forest therapy programs. The control group, which continued pharmacological therapy, also showed a decrease in stress levels, but this change was not statistically significant. The mean baseline score for the experimental group was 22.88 points (“severe”), which decreased to 18.94 points (“moderate”) following the program. In the control group, the mean score decreased from 21.33 to 20.25, remaining in the “severe” category. These findings suggest that participation in the forest therapy programs contributed to a reduction in stress levels. Previous research has demonstrated that forest therapy programs are associated with a significant decrease in stress levels. For instance, Kweon et al. (27) reported a reduction in stress levels among individuals from various professional backgrounds, including healthcare providers, IT specialists, and teachers, following a forest therapy program. Guo et al. (28) observed that forest walks alleviated stress and promoted physical and mental recovery in 247 university students. Yang et al. (29) found that a forest therapy program incorporating sensory-based activities reduced stress in university students, with women experiencing greater stress reduction than men. Systematic reviews (30–32) and meta-analyses (33–35) have further indicated the efficacy of these methods.

Second, the analysis of the negative and positive perception subdomains of the PSS revealed that only the positive perception score decreased significantly in the experimental group. In this group, negative perception scores decreased from 9.71 to 7.82, but this change was not statistically significant. The mean score for both negative (10.83 to 10.25) and positive perception (10.50 to 10.00) decreased in the control group, but neither change was statistically significant. The significant decrease in positive perception scores in the experimental group suggests a positive shift in participants’ ability to recognize and cope with stress following forest therapy. The negative perception subdomain reflects anxiety and helplessness in response to stress. Although the experimental group’s scores decreased, the change was not significant. These findings suggest that the personalized forest therapy programs were more effective in helping the participants cope with stress and perceive stress positively than in reducing anxiety stemming from negative perceptions of stress and a sense of lack of control.

Previous research examining the effects of negative and positive perceptions of stress remains somewhat limited. Lee et al. (36) reported that, in cancer patients, total stress scores decreased after forest therapy, but negative perception scores increased, indicating no improvement in that subdomain. The present study’s finding of a significant decrease in positive perception subdomain scores was interpreted as being related to stress-coping methods. This finding aligns with studies showing that forest-based exercise (37), meditation and self-exploration in a forest environment (38), and a self-development program (39) positively influence stress coping strategies. Collectively, these prior studies indicate that forest therapy programs provide opportunities for participants to develop stress-coping skills and better self-management capacity.

These findings also support the conceptualization that changes in positive and negative perceptions operate as independent dimensions, corresponding to the dual-continua model of wellbeing and ill-being rather than a single bipolar continuum (40). The absence of significant changes in negative perception scores suggests that ill-being components—such as stress and anxiety—may possess relative stability and are less likely to be reduced through short-term interventions. In contrast, the significant reduction in positive perception scores can be interpreted as evidence that the participants engaged in problem-solving, emotional processing, and reflective activities throughout the program, thereby activating problem-focused coping strategies. This interpretation aligns with previous research demonstrating strong associations between positive emotional appraisal and problem-focused coping (24).

Taken together, the findings of this study reinforce the theoretical perspective that wellbeing and ill-being change independently and that improvement in one domain does not automatically translate into improvement in the other (41). Furthermore, individuals with depression may continue to experience negatively appraised stress while still retaining the capacity to restore and enhance positive emotional functioning—a core aspect of wellbeing. Therefore, incorporating components that foster positive emotional experiences may play a critical role in enhancing the effectiveness of future forest therapy programs.

Third, regarding obsessive-compulsive symptoms, the experimental group demonstrated a decrease in scores, while the control group exhibited an increase. Although these changes were not statistically significant, the experimental group had a better result than the control group. Both groups’ pre- and post-test scores fell within the “normal” range based on the MOCI scale; nevertheless, this scale was originally validated in college students and thus may not be entirely suitable for the current study’s sample of adults aged 20 to 50. The mean pre-intervention score in the experimental group decreased from 43.82 to 42.59, while that of the control group increased from 41.92 to 42.50. Although the range remained normal, the downward trend in the experimental group suggests potential benefits. These results align with a previous study showing that forest therapy activities reduced obsessive-compulsive levels in menopausal women (42), as well as another study indicating that obsessive-compulsive symptoms decreased after participating in forest therapy programs eight or 12 times (43). Although many other therapeutic approaches, including art therapy (44), cognitive behavioral therapy (45), and play therapy (46), have been shown to alleviate obsessive-compulsive symptoms, interventions in forest environments remain underexplored. While the current study did not demonstrate a statistically significant decrease in obsessive-compulsive symptoms, a certain level of change was observed, highlighting the need for further research on the effectiveness of forest therapy in alleviating these symptoms.

Fourth, this study implemented the program in an accessible urban forest to enhance the participants’ engagement and continuity in forest therapy. Although the “forest” environment is a critical therapeutic component, many individuals experience psychological or logistical burdens when traveling to remote forest locations. While the sense of “escape” that comes from leaving urban settings can be therapeutically valuable, forest therapy programs must also be delivered in easily accessible nearby urban forests or neighborhood parks within walking distance (47). Considering the sustainability of forest therapy participation, providing programs in locations that can be incorporated into daily routines may increase patients’ voluntary visitation frequency. This is consistent with previous findings that proximity to green spaces is positively associated with the frequency of use (48). Consequently, recent research has increasingly utilized urban forests to overcome accessibility barriers, and further studies in this area are warranted.

This study also underscores the value of personalized forest therapy by identifying program elements appropriate for each patient through collaboration with healthcare professionals. Attending specialists completed a diagnostic evaluation form that included questions regarding appropriate therapy activities and programs for each patient, as well as considerations for the forest therapy instructor to keep in mind during program development and implementation. This process enabled specialists with limited prior knowledge of forest therapy to participate in the design of the programs. Tailored programs were then developed accordingly. Given the ongoing need for forest therapy programs tailored to participants (17, 49, 50), this study provided an opportunity to develop personalized forest therapy programs and investigate their effectiveness in reducing stress and obsessive-compulsive symptoms. We hope that, in the future, a system will be developed through which forest therapy programs can be delivered and applied, with each patient receiving personalized activities and guidance directly from their attending specialist.

Despite this study’s promising results, there are some limitations that should be acknowledged. First, the number of participants in this study was 29, which could be considered a small sample size. Consequently, it is challenging to generalize the findings of this study to a broader population. Future research should consider increasing the sample size to ensure the representativeness of the results. Second, both the experimental and control group participants were receiving hospital treatment. However, the specific dosage or frequency of the medication being taken was not known. Subsequent studies should account for these potential confounding variables. Third, the study did not fully control for incidental exposure of the control group to natural environments in daily life, such as parks or urban forests. Future studies should consider controlling for this factor. Fourth, this study provided the experimental group with personalized forest therapy programs but did not directly compare them to standard, non-personalized forest therapy programs. Future studies should compare personalized forest therapy programs with standardized forest therapy programs.

Despite these limitations, this study demonstrated that personalized forest therapy programs, designed in collaboration with attending specialists, contributed to reducing stress and obsessive-compulsive symptoms in the patients with depression. These findings suggest that such programs hold promise as a form of non-pharmacological therapy. Future expansion of healthcare collaboration could establish a foundation for utilizing forest therapy programs as non-pharmacological interventions in clinical settings.

## 5 Conclusion

This study demonstrated that personalized forest therapy programs effectively decreased stress and obsessive-compulsive symptoms in the patients with depression. These findings suggest that forest therapy programs tailored to individual patient characteristics may contribute to improving the mental health of patients with depression. With the future expansion of collaboration between forest therapy practitioners and the healthcare sector, integrating personalized forest therapy programs alongside pharmacological and counseling therapies is expected to have a positive impact on alleviating stress and obsessive-compulsive symptoms in patients with depression.

## Data Availability

The datasets presented in this article are not readily available because the dataset contains sensitive clinical and psychological information from patients with depression. Due to privacy and ethical restrictions, individual-level data cannot be shared publicly or upon request. Only aggregated results are available within the manuscript. Requests to access the datasets should be directed to Gayeon Kim, rkdus6520@naver.com.
